# Cellular adaptations leading to coral fragment attachment on artificial substrates in *Acropora millepora* (*Am-*CAM)

**DOI:** 10.1038/s41598-022-23134-8

**Published:** 2022-11-01

**Authors:** Brett M. Lewis, David S. Suggett, Peter J. Prentis, Luke D. Nothdurft

**Affiliations:** 1 School of Earth and Atmospheric Sciences, Faculty of Science, Queensland University of Technology, Brisbane, QLD Australia; 2 Climate Change Cluster (C3), University of Technology Sydney, Sydney, NSW Australia; 3 Centre for Agriculture and Bioeconomy and School of Biology and Environmental Sciences, Faculty of Science, Queensland University of Technology, Brisbane, QLD Australia

**Keywords:** Animal behaviour, Animal physiology, Biomechanics, Autophagy, Cell growth, Cellular imaging, Cytoskeleton, Cell growth, Cell proliferation, Differentiation, Stem cells, Cell biology, Developmental biology, Evolution, Immunology, Zoology, Microbiology, Biofilms, Physiology, Bone, Reproductive biology

## Abstract

Reproductive propagation by asexual fragmentation in the reef-building coral *Acropora millepora* depends on (1) successful attachment to the reef substrate through modification of soft tissues and (2) a permanent bond with skeletal encrustation. Despite decades of research examining asexual propagation in corals, the initial response, cellular reorganisation, and development leading to fragment substrate attachment via a newly formed skeleton has not been documented in its entirety. Here, we establish the first "coral attachment model" for this species ("Am-CAM") by developing novel methods that allow correlation of fluorescence and electron microscopy image data with in vivo microscopic time-lapse imagery. This multi-scale imaging approach identified three distinct phases involved in asexual propagation: (1) the contact response of the coral fragment when contact with the substrate, followed by (2) fragment stabilisation through anchoring by the soft tissue, and (3) formation of a "lappet-like appendage" structure leading to substrate bonding of the tissue for encrustation through the onset of skeletal calcification. In developing Am-CAM, we provide new biological insights that can enable reef researchers, managers and coral restoration practitioners to begin evaluating attachment effectiveness, which is needed to optimise species-substrate compatibility and achieve effective outplanting.

## Introduction

Coral reef development begins when new reef-building coral attach to favourable substrates via planulae settlement or the (re-) attachment of fragments that have broken from adult colonies via physical disturbances^1–3^. As a result, discerning the biological adaptations required to establish attachment is a principal factor for understanding how coral reefs initiate, grow and recover post-disturbance. However, the biological processes required for effective coral fragment attachment to substrates remain largely unknown despite an ongoing need to establish the fundamental biology of attachment to progress decades of research focused on coral growth^4–10^ and reef development^11–15^.

Colony fragmentation through physical disturbances is a common part of a corals day-to-day life due to tidal flows, waves and frequent storms^16–18^, which biological factors may also aid (e.g., bioeroders^19^). Fragments that successfully adapt to form an attachment can go on to form adult colonies through asexual reproduction^1,2,16^and begin reproducing and contributing to coral populations within 1–2 years^1,20^. To date, fragment attachment has broadly been defined as “the first basal tissue” growing onto a substrate, with success quantified as the extent of basal tissue visible at the coral-substrate interface^21,22^. While these broad definitions as to what constitutes attachment have been useful for comparing survivorship, this description does not consider the different phases of the coral attachment process or the degree to which the fragment is “attached”; namely, whether the attachment consists of only soft tissues or whether a new skeleton is present on the contact surface.

Successful attachment can be inhibited by biological and environmental factors such as substrate type^23,24^, substrate mobility and water movement^25–27^. Several organismal and external processes are suggested to play a role in the effective attachment of a coral fragment. These include (1) a contact response to promote growth and protect the colony against pre-existing debris, organisms and/or pathogens on the substrate^28–32^; (2) passive stabilisation of the fragment via tissue anchoring, interlocking with the substrate or low water currents to reduce soft tissue damage due to constant movement^33^; (3) maintenance of extracellular matrices (ECM), which facilitates cell–cell and cell–substrate adhesion^34–36^; and/or (4) the transition from the outer surface body wall (SBW) into a basal body wall (BBW) possessing the specialised cells required for ECM production and the controlled deposition of a calcium carbonate skeleton^6,7,37–39^. However, if or how these various factors play a role in the attachment process has not been evaluated. Such uncertainty reflects, in part, the difficulty in observing such precise micro-anatomical developments simultaneously and at multiple scales.

Live and static imaging is an effective way to detail the structures of reef-building corals at the organismal, tissue and cellular levels^6,38,40–45^. Recent advancements in imaging techniques have provided vital insight into coral calcification, biomechanics and cellular plasticity^38,40–43^, including non-invasive techniques for dynamic temporal imaging at high resolution, such as in vivo video and time-lapse microscopy^46–48^ and novel static imaging^41–43^. The use of in vivo observations is maximised when used in combination with static microscopy to increase the resolution of observation in characterising soft tissue and skeletal development^6,43,49^. However, sample preparation for the majority of static microscopy used in coral anatomy compromises the three-dimensional analysis of the soft tissues and skeleton by relying on one of two processes for sample preparation; (1) physical or chemical removal of the soft tissue to expose the skeleton or (2) decalcification of the skeleton leaving behind only fixed tissues^5,50–52^. As a result, imaging of both the coral skeleton and coral soft tissues together is rare^42,53^. Investigating both the soft tissues and skeleton simultaneously can significantly advance knowledge of fundamental coral biology and help us understand the innate adaptability of corals, which is considered an important “first step” for all coral conservation initiatives^42^.Existing approaches to employ coral outplanting as part of reef intervention management tools have suggested that the durability of attachment dictates coral fragment success^22,24,54^ and that success can be improved with a stable point of contact between the coral fragment and the substrate^21,23^. To date, one study^22^ has examined fragment attachment processes within the first month of deployment. Such paucity of observations of coral attachment through the initial stages of contact obscures the systematic nature by which the coral fragment changes from the time of substrate contact; hence the tissue and skeletal processes and factors that promote or inhibit rapid and robust attachment remain unknown. If aquaculture and outplanting via fragment re-attachment are to be viable for targeted reef restoration^55,56^, it is vital to assess coral attachment at the appropriate spatial and temporal resolution. As such, using novel in vivo approaches at multiple temporal and spatial scales, we sought to model the biological adaptations required for reef-building coral *A. millepora* to build a skeletal attachment at the substrate-to-fragment interface to understand settlement and attachment processes.

## Results

**Figure 1:**
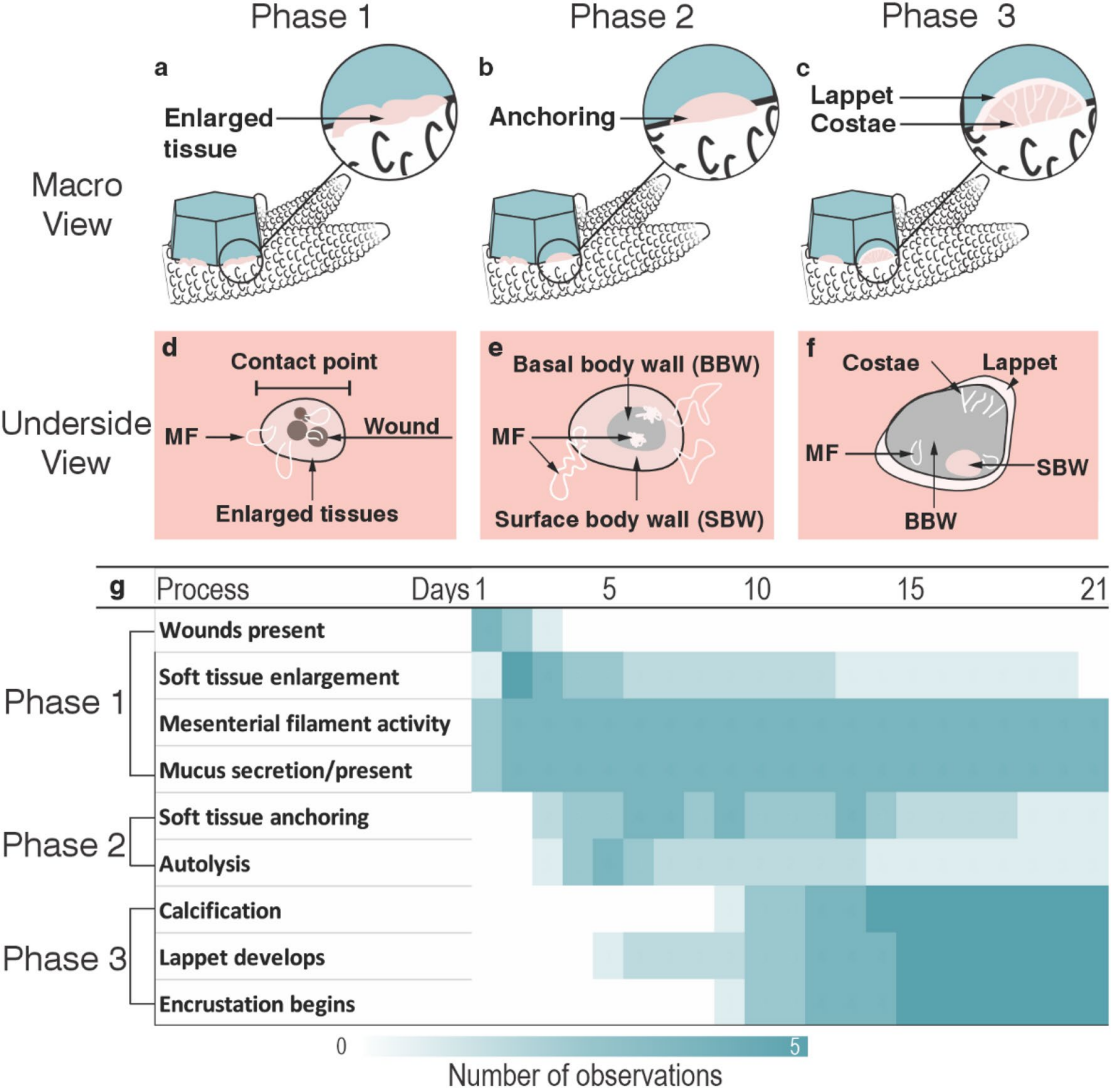
An overview of the three phases of attachment over 21 days from both the expanded view and the obscured underside and with the timing of each stage and their characteristics. (**a**–**c**) Coral attachment through three stages: (1) contact response leading to (2) soft tissue anchoring and fragment stabilisation and (3) calcification and lappet-like appendage development leading to bonding and encrustation. (**e**, **f**) Micro view of the underside in all three stages. (**h**) The timing of the processes which characterise each phase represented by a heat map.

Three distinct phases of morphological changes and cellular and tissue remodelling were observed during fragment attachment (Fig. 1): phase one: contact response preparing for fragment attachment—0–5 days (Fig. 1a); phase two: fragment stabilisation through soft tissue anchoring—3 to 8 days (Fig. 1b); and phase three: lappet-like appendage development and calcification leading to permanent bonding and encrustation—5 to 12 days (Fig. 1c). Each phase initiated sequentially across all contact points and for all samples (i.e., phase 2 never began before phase 1, etc.), where the complex nature of fragment architecture resulted in multiple contact points that cannot be controlled for. Each contact point exhibited spatial and temporal variations in phases and phase characteristics. Detailed characterisations of the of the various phases are as follows:

### Phase one: contact response

#### Gross anatomy

**Figure 2:**
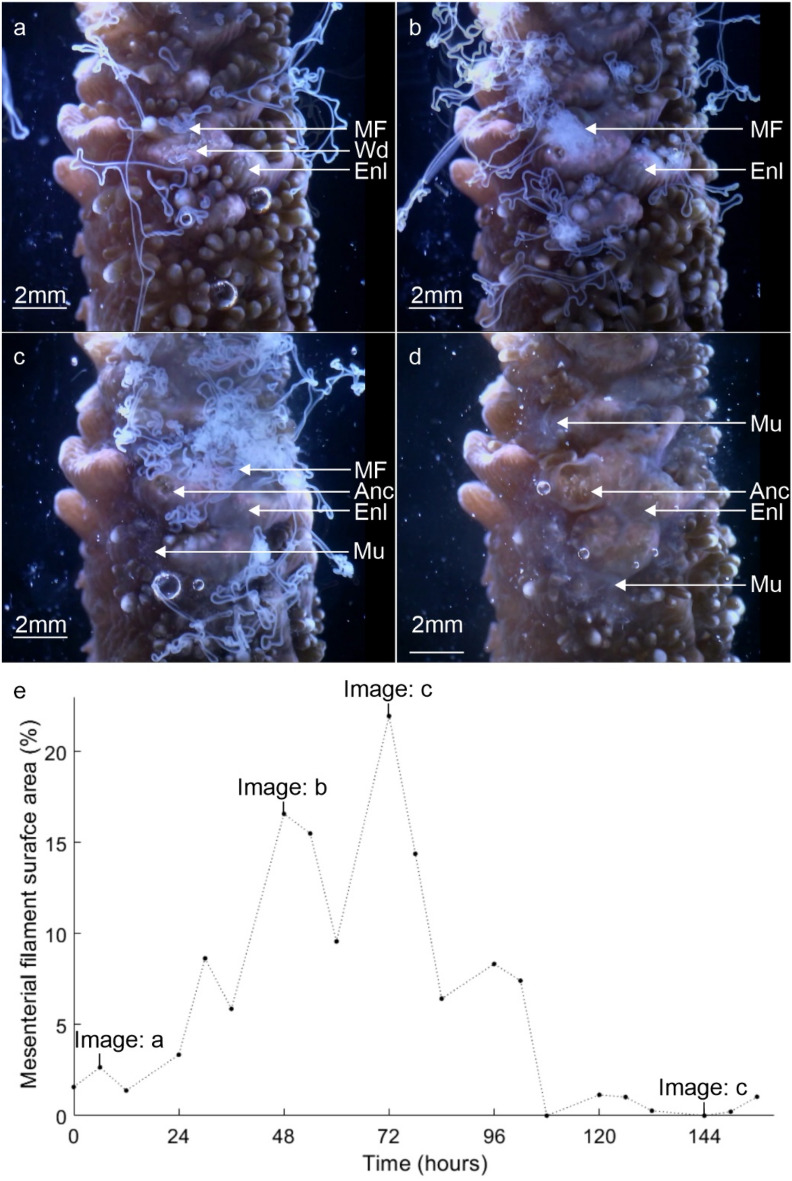
Stills from time-lapse light microscope movies displaying the representative processes that characterise the contact response phase, i.e., tissue enlargement, mucus release, wound cleaning (0–5 days). (**a**) Wounds (Wd) formed by abrasion are cleaned by mesenterial filaments (MF) as the surrounding immunocompromised tissues become enlarged (Enl). (**b**, **c**) Mesenterial filament deployment is increased external to the body cavity as the compromised tissues become further enlarged (2–3 days). (**d**) Mucus (Mu) secretions that have formed on the substrate surface surrounding the compromised tissues change colour over time—from transparent to white or brown. (**e**) A plot showing the relative density of the mesenterial filaments activity inside and outside the body cavity on each day the still (a,b,c,d) was captured. Wound; Wd, Enlarged tissues; Enl, mesenterial filaments; MF, mucus layer; Mu.

First contact between the coral fragment and the substrate produced wounds in soft tissues via abrasion (Fig. 2a). The soft tissues then underwent a contact response, which lead to the wound healing over the first 1 to 3 days and the cellular reorganisation and development required for the transition into the second phase of attachment. The contact response lasted 3–5 days.

**Figure 3:**
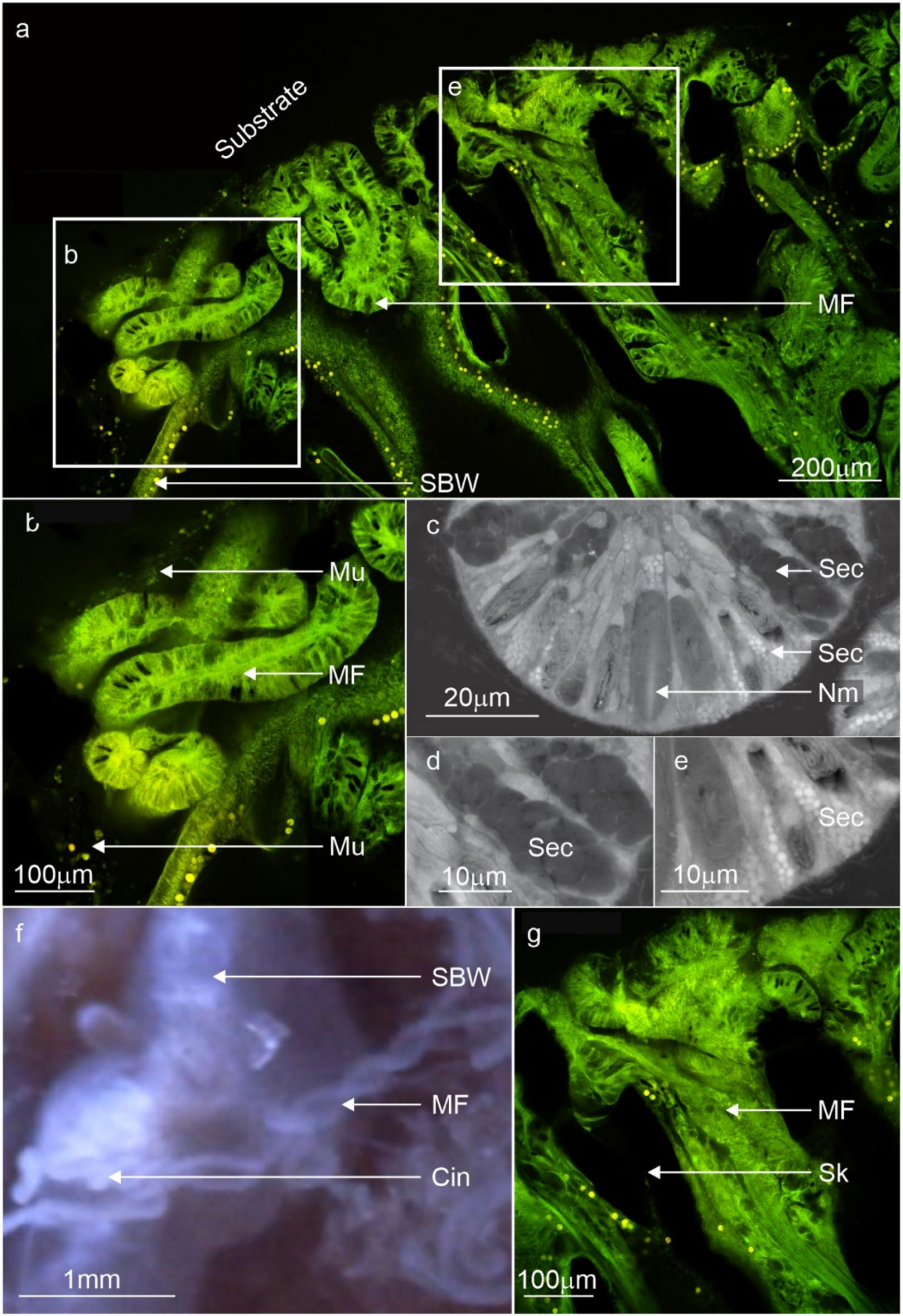
Confocal fluorescence microscopy images, backscatter electron microscopy images and stills from time-lapse microscopy movies highlighting the distribution, cell composition and mesenterial behaviour during the contact response phase. (**a**, **b**) Contact with the substrate lead to the deployment of mesenterial filaments (MF) in newly formed wounds and the formation of a mucus barrier. (**c**–**e**) A diverse number of secretory cells (Sec) were present in the mesenterial filaments of *A. millepora*, and their proximity to the wounds and mucus indicates a role in cleaning and mucus formation. (**f**) For mesenterial filaments to move through the SBW and extend beyond the body cavity, they employ a twisting or corkscrewing motion to slide out of the cinclide-like (Cin) openings (Supplementary Movie S2). (**g**) Fluorescence image of the mesenterial filament (MF) twisting through the cinclide-like structure and the costae (Sk). surface body wall; SBW, mucus layer; Mu, Nm; nematocyst, Skeleton; Sk.

The surface body wall (SBW) around the wounds (and in contact with the substrate) became enlarged (approx. 100–150 µm compared to the coenosarc SBW approx. 40 µm) (Fig. 2b). Localised mucus excretions occurred at the contact points, most of which eventually dissolved into the water column. However, some mucus remained as fibrous strands or as a layer on the substrate surrounding the contact tissues (Fig. 2c) that, over time, became more visible turning from transparent to white and eventually brown (Fig. 2d). This mucus layer was more durable and remained until it was physically dislodged. A significant increase in mesenterial filament activity within the tissue wounds and external to the body cavity occurred during the first 5 days (Fig. 2e). To gain access to the surrounding environment, the mesenterial filaments build-up at the underside or distal edge of the coral fragments SBW. Here, a temporary ‘cinclide-like’ opening develops in the SBW through which multiple mesenterial filaments extend using a spiralling ‘corkscrew’ motion (Fig. 3a,f,g) (Supplementary Movie S2). Time-lapse imagery documented the biological processes that underlie tissue recovery, substrate sterilisation and foreign material removal—all processes driven by changes in the cells and tissues.

#### Micro anatomy

**Figure 4:**
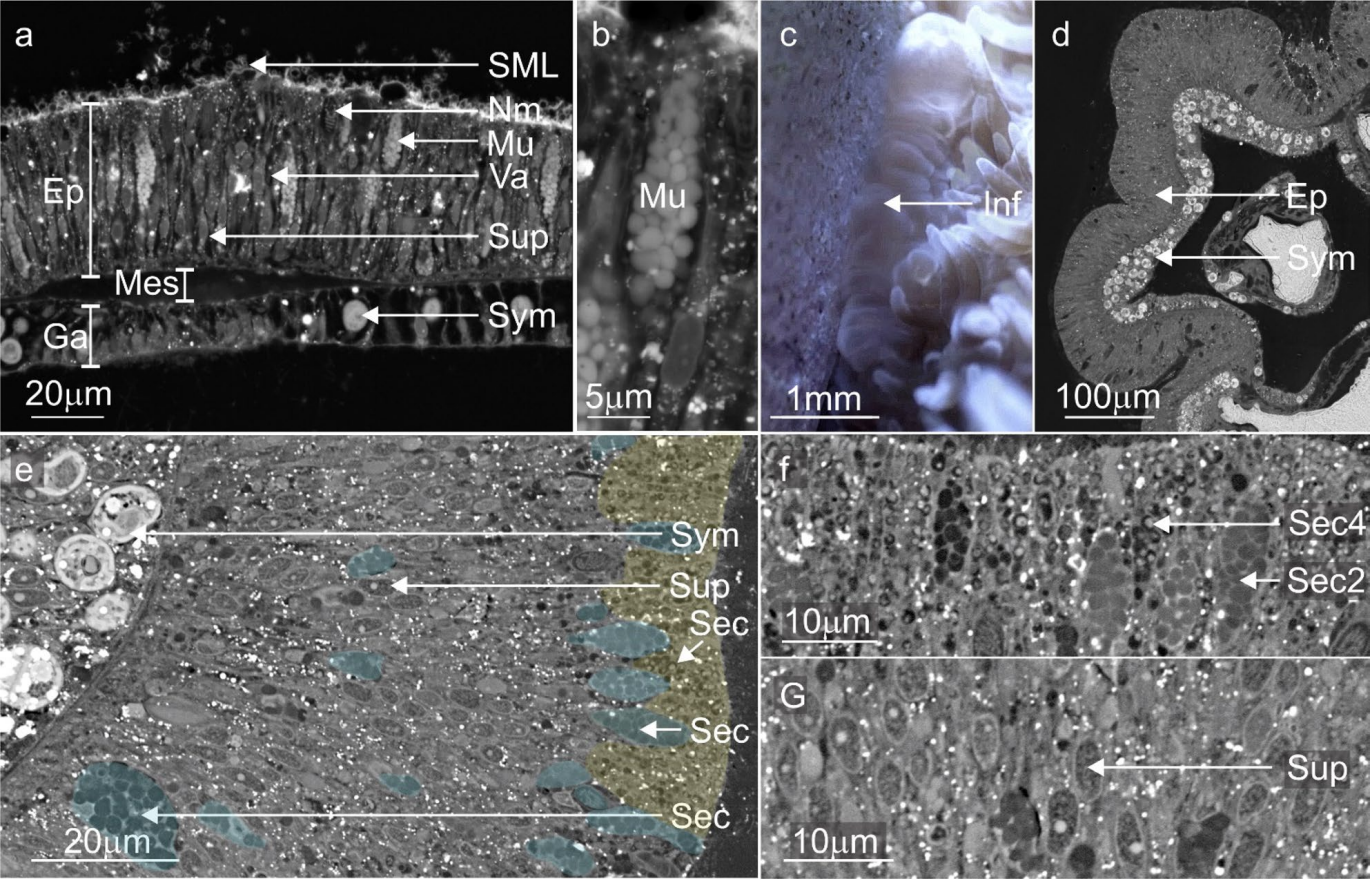
Backscatter electron microscopy images and stills from time-lapse movies comparing *A. millepora' 's* regular SBW with the enlarged tissues during the contact response phase. (**a**) A standard surface body wall (SBW) of *A. millepora* showing the two epithelial layers (epidermis; Ep, gastrodermis; Ga) separated by the mesoglea (Mes) and the cells and processes that characterise these layers; supporting cells (Sup), intercellular vacuole (Va) or spaces in between each cell, nematocysts (Nm), type 2 gland cell (Mu), Symbiodiniaceae (Sym) and the soluble surface mucus layer (SML). (**b**) Higher magnification image of the vesicles (white) in standard type 2 gland cells (mucocytes). (**c**, **d**) The soft tissue became enlarged at the points of contact between the coral and the substrate. (**d**) Electron microscopy images of the enlarged SBW tissues showed a proliferation of densely packed thin supporting cells and Symbiodiniaceae. and the differentiation/proliferation of type 4 (yellow) and type 1 and 2 (blue) gland cells (Sec) with lead to the loss of intercellular vacuoles. (**e**, **f**) Electron image showing the vesicles (Sec4) of type 4 gland cells with matter trapped inside and mucocytes (Sec2). (**g**) Closer look at the densely packed epitheliomuscular supporting cells (Sup) and their nuclei. Skeleton; Sk, surface body wall; SBW, epidermis; Ep, gastrodermis; Ga, mesoglea; Mes, surface mucus layer; SML, Nm; nematocyst, supporting cells; Sup, intercellular vacuole; Va, secretory cells; Sec, type 2 gland cell; Mu and Sec2, type 4 gland cell; Sec4, Symbiodiniaceae; Sym.

The proximal and temporal relationship between the durable mucus and mesenterial filaments observed during time-lapse microscopy (gross anatomy) was supported by autofluorescence imaging. Images show the mesenterial filaments were external to the body cavity, in-between the substrate and the developing mucus biofilms (Fig. 3a,b). Histological investigation of the mesenterial filaments also confirmed that the cnidoglandular band of the mesenterial filament possesses numerous secretory gland cells (Fig. 3c,d,e), capable of producing variations in mucus development^57^ and digesting matter^58^. The enlarged tissues observed during time-lapse, when cross-sectioned and then imaged with the SEM (Fig. 4), showed a rapid proliferation of supporting cells (Fig. 4e,g) and three distinct morphologies of secretory gland cells in the epidermis (Fig. 4e,f) and algal Symbiodiniaceae in the gastrodermis (Fig. 4e).

### Phase two: soft tissue anchoring and fragment stabilisation

**Figure 5:**
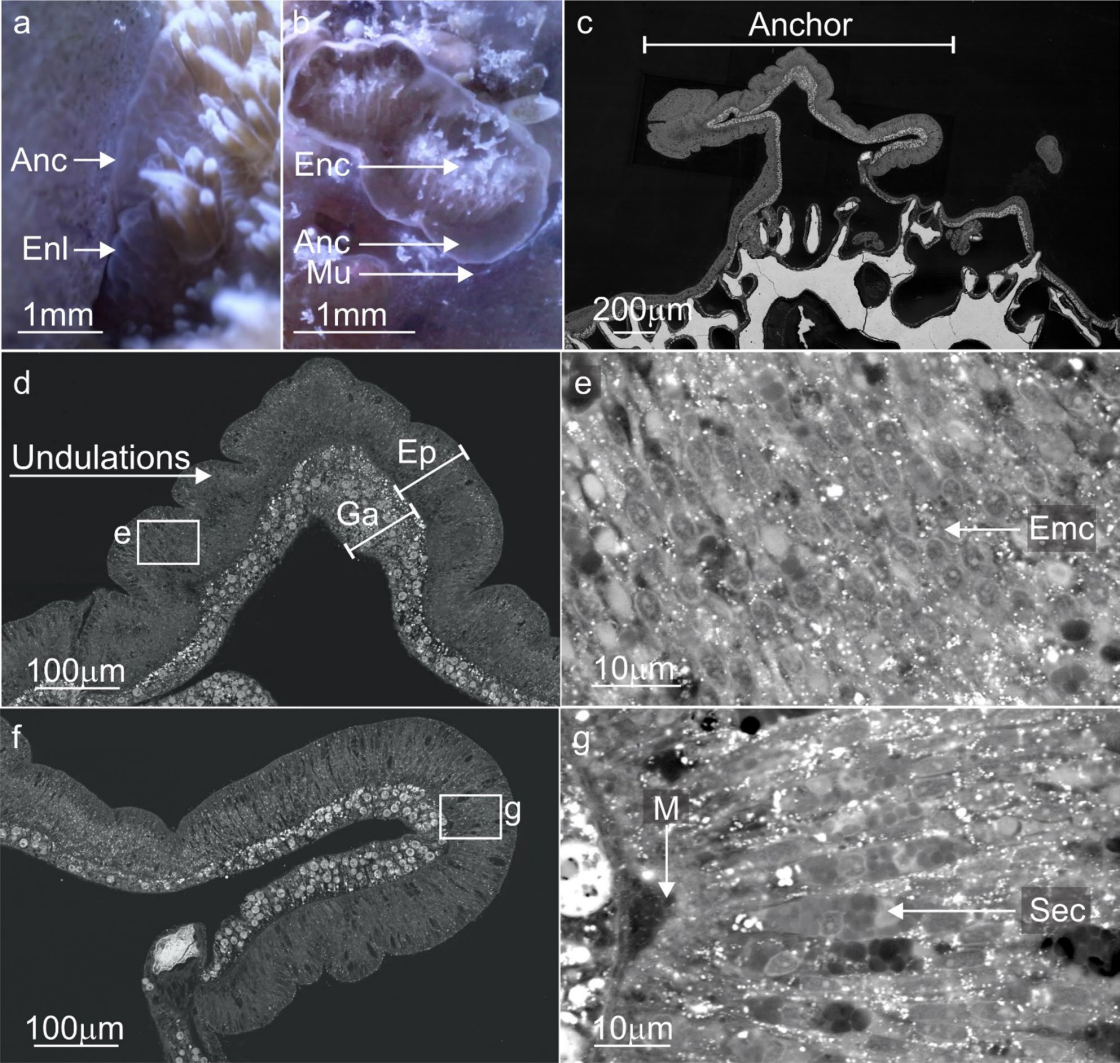
Backscatter electron microscopy images and stills from time-lapse microscopy movies comparing *A. millepora' 's* regular tissues with the enlarged anchored tissues. (**a**) The enlarged tissues (Enl) from phase one develop into a that anchors and helps stabilise the fragment. (**b**, **c**) The anchoring process creates a sealed or enclosed (Enc) environment where the SBW or epidermis can be safely removed and a BBW can form. (**d**, **e**) The anchoring tissue took on a complex undulated morphology that likely assists tissue anchoring. The shifting morphology of these tissues is down to an ongoing proliferation of supporting cells which also act as epitheliomuscular cells (Emc) and (**f**, **g**) gland cells (Sec), which may also aid with adhesion. Enlarged tissues; Enl, soft tissue attachment; Anc, enclosed environment; Enc, epidermis; Ep, gastrodermis; Ga, mesoglea; Mes.

In phase one, soft tissues underwent localised wound healing, cell proliferation and mucus release at the contact point (phase one) to promote recovery and growth of the SBW. As a result, the tissues at the contact interface enlarged, increasing musculature and morphological complexity and contact surface area of the tissues. Along with isolated tissue pulsing, the change in cell size and morphology aided tissue expansion, surface “grip”, and as a result, active stabilisation of the fragment (Supplementary Movie S3) (Fig. 5a,b). The proliferation of the supporting cells, which function as epitheliomuscular cells, enabled the biomechanics for pulsing via the expansion and growth of fragment cytoskeleton, which also led to the development of the complex tissue morphology (Fig. 5c,g). The anchored tissues formed an enclosed, sealed area where the SBW transitioned into a BBW that produced the extracellular matrices and a calcium carbonate (CaCO_3_) skeleton. Anchoring occurred 3 to 12 days after substrate contact.

#### Gross anatomy

**Figure 6:**
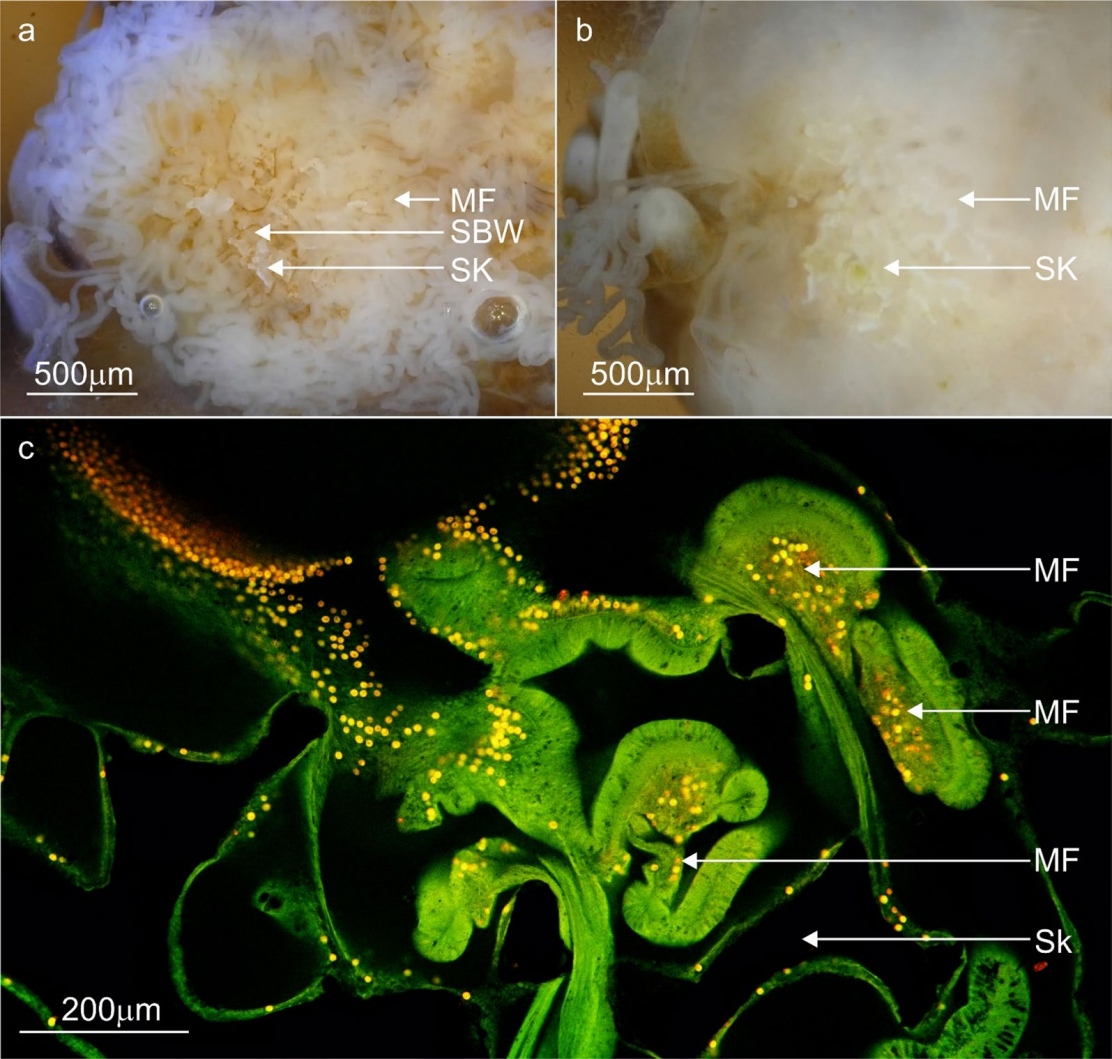
Representative confocal fluorescence microscopy images and stills from time-lapse microscopy movies highlighting the distribution, cell composition and mesenterial behaviour during the contact response phase. (**a**) The fragment deploys mesenterial filaments (MF) *en-masse* as concentrated balls to begin removing the epidermis of the enclosed SBW of the anchored tissue in contact with the substrate. (**b**) The removal of epidermal cells leads to the development of cells needed for skeleton precipitation. (**c**) This image shows the mesenterial filaments in the anchored or compromised tissues with increased numbers of Symbiodiniaceae in the mesentery filaments which could be a sign of their digestion. Skeleton; Sk, mesenterial filament; MF, surface body wall; SBW.

The soft tissues continue to develop from phase one until they became anchored to the substrate (Fig. 5a) (Supplementary Movie S3), thereby stabilising the coral fragment to the substrate and initiating phase two. Stabilisation by anchoring produced a relatively weak attachment, as evidenced by the retracting or detaching of the anchored tissues during the deployment of large numbers of mesenterial filaments or during slight movements of the substrate or fragment. In some cases, anchoring is initiated at multiple contact points in an individual fragment. However, the rate and degree of anchoring varied among the contact points, with some points seemingly delaying development until other contact points reached the bonding stage (phase three). The anchored tissues inherently created an enclosed space at the interface between the tissue and the reef substrate. A systematic deployment of mesenterial filaments began once the sealed space was created, which removes the enclosed SBW via autolytic processes (Fig. 6) (Supplementary Movie S4). Only after this systematic deployment of filaments did skeletal precipitation begin at these sites (from 9 to 14 days). No evidence of skeleton production before autolysis was observed in any samples.

#### Micro anatomy

The continued proliferation of supporting cells increased the musculature and morphological complexity of the soft tissues at the contact interface, which in turn, increased localised pulsing and the development of the soft tissue anchor observed in the time-lapse. Secretory gland cells (including mucocytes) also continued to proliferate in soft tissues at the contact interface. The mesenterial filaments removed the enclosed tissues/epidermis of SBW and any superfluous cells (including Symbiodiniaceae) via autolysis (Fig. 6). The space created allowed for the development of the calicodermis on the remaining gastrodermal layer, completing the transition from a SBW to a BBW required for ECM development and calcification (Fig. 6). Vestigial epidermal cells (discussed in phase three) from the original epidermis were occasionally present in the newly developed calicodermis (Fig. 8a). Mesenterial filaments contained several uncharacterised secretory cells as identified by SEM (Fig. 3c,d,e).

### Phase three:  calcification and the development of a lappet-like appendage leading to substrate encrustation

#### Gross anatomy

**Figure 7:**
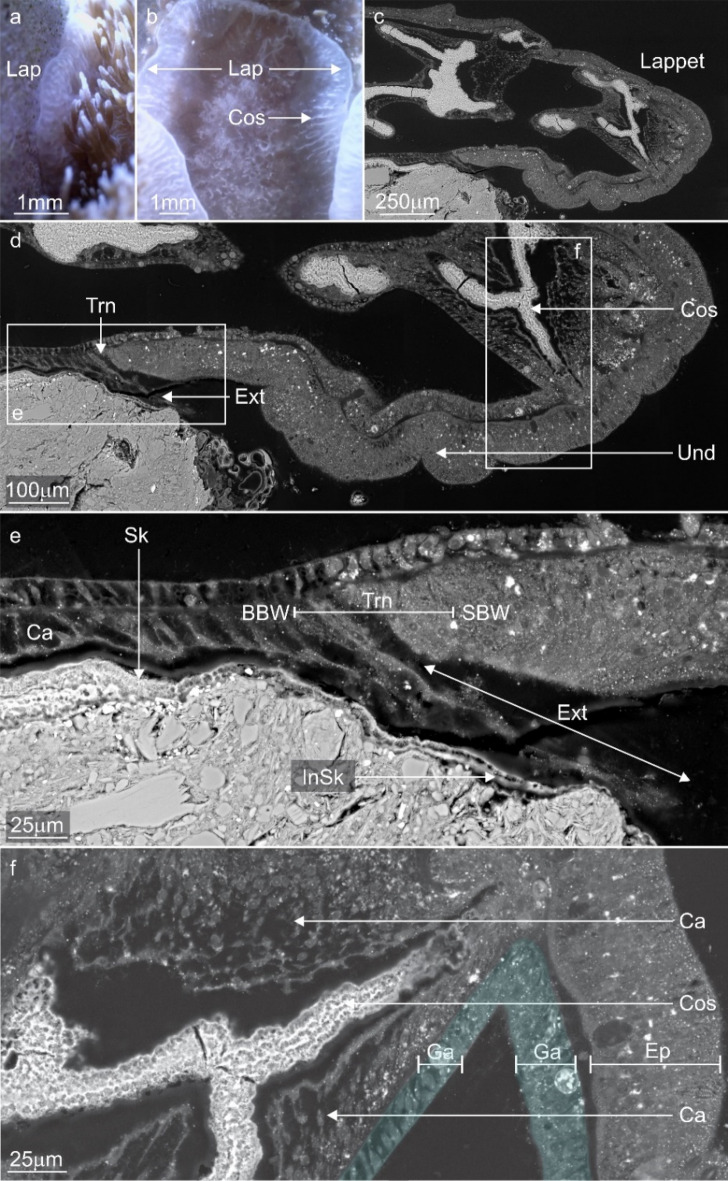
Backscatter electron microscopy images and stills from time-lapse microscopy movies highlighting the location, morphology and function of the lappet-like appendage vital for encrustation of the substrate and for forming an enduring bond. (**a**, **b**) The lappet-like appendage (Lap) is located on the distal edge of the encrusting rim and is responsible for both initial basal precipitation and costae development (Cos). (**c**) The lappet-like appendage is a complex structure possessing an SBW that is thicker than the coral regular SBW. (**d**) Higher magnification view of the lappet-like appendage shows a complex morphology consisting of 4 key characteristics: (1) tissue undulations (Und) with densely packed cells on its underside, (2) a transition zone (Trn) where the SBW transitions into the BBW, (3) a '''pocket' of calicoblastic cells (Cos) for costae development (Cos)—this is not always present as some areas are not actively precipitating costae and (4) a thin, poorly defined continuation of the calicoderm (Ext) that sits between the lappet-like appendage and the substrate surface and is responsible for the first layers of skeleton (InSk). (**e**) Higher magnification view of the transition zone (Trn) and the poorly defined calicoderm extension (Ext). Calicoderm extension is non-traditional in that it does not possess a gastrodermis directly adjacent to the corals, which is the standard tissue arrangement. Both epithelial layers of the SBW primarily consist of supporting epitheliomuscular cells, while the BBW consists of a standard calicodermis and gastrodermis. As the lappet-like appendage slowly migrates, it leaves behind a trail of new BBW to continue colony growth and skeletal thickening. (**f**) The '''pocket' of cells responsible for the costae wall (Cos) forms between the lappet-like appendage SBW epidermis (Ep) and gastrodermis (Ga) (Blue) at the mesoglea. skeleton; Sk, epidermis; Ep, gastrodermis; Ga, calicodermis; Ca, undulations; Und, transition zone; Trn, calicodermis extension; Ext.

A lappet-like appendage, superficially similar to a lappet appendage in epithecate corals, developed at the distal edge of the anchored tissue of *A. millepora*. This lappet-like appendage appears discoloured or lighter than the surrounding tissues, is polyp free and enlarged compared to the standard coenosarc (Fig. 7a,b). The lappet-like appendage was capable of systematic pulsed contractions or undulations that circulated around the encrusting rim where the lappet-like was present (Supplementary Movie S5).

#### Micro anatomy

**Figure 8:**
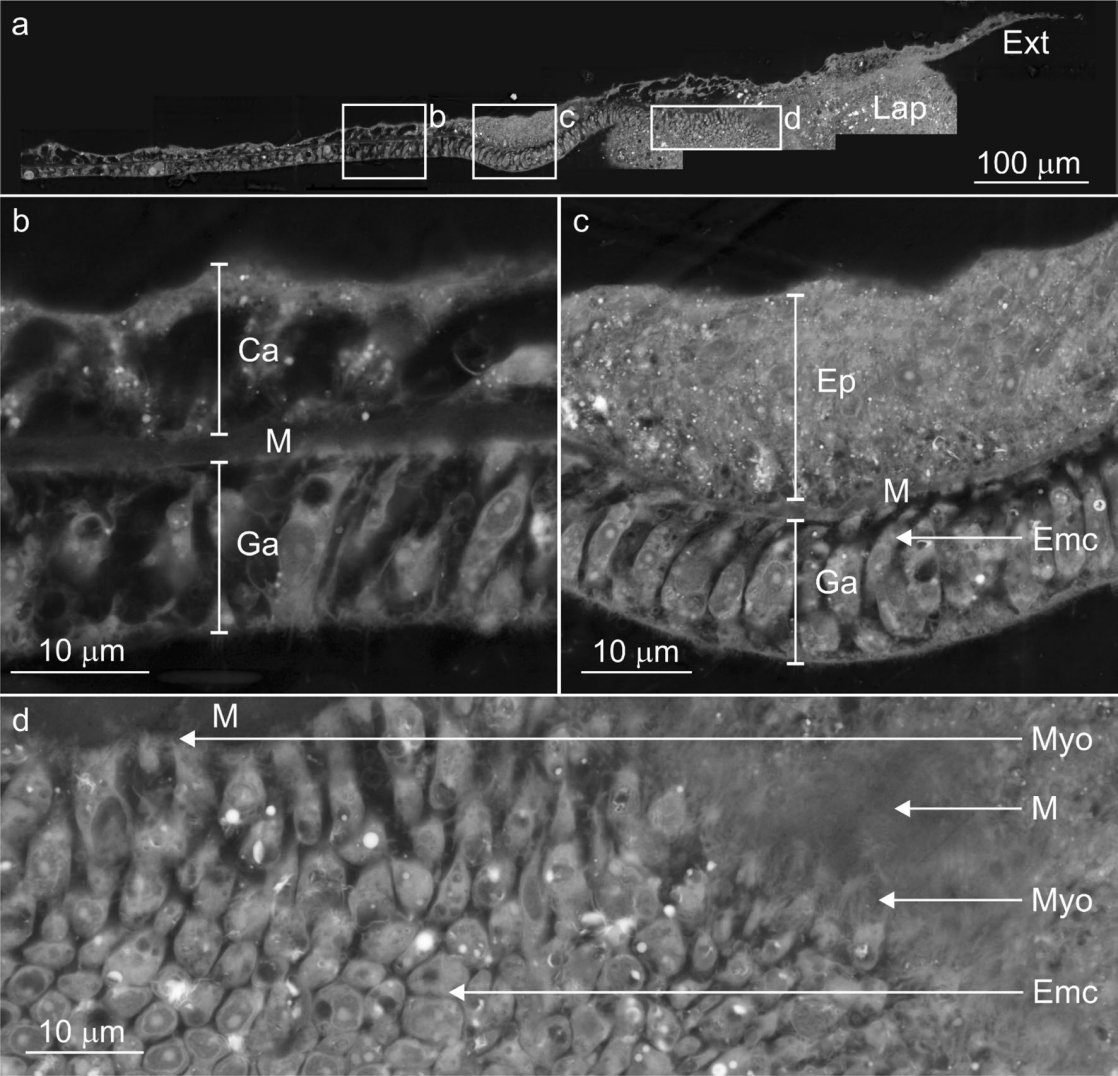
Backscatter electron microscopy images highlighting the primary cell composition of a newly developed lappet-like appendage. (**a**) The base of the developing lappet-like appendage (right) and the trailing basal body wall (BBW, left). (**b**) The standard BBW consists of two epithelial layers separated by the mesoglea (M); the calicodermis (Ca) primarily consists of cuboidal calicoblasts and a gastrodermis (Ga). (**c**) A pocket of vestigial epidermal cells that has not yet been removed by mesenterial filaments is still present in the BBW trailing the new lappet-like appendage. (**d**) The lappet-like primarily consists of epitheliomuscular cells (Emc) that attach to the mesoglea (M) by their filamentous myofibrils (Myo). The heightened musculature gives the lappet-like its complex undulated morphology and the mechanical ability to pulse and perhaps grip the surface. Lappet-like; Lap, costae; Cos, surface body wall; SBW, epidermis; Ep, transition zone; Trn, calicodermis extension; Ext, epitheliomuscular supporting cells; Emc.

Tissue cross-sections identified that 4 distinct features characterise the lappet-like appendage: (1) a larger than usual surface body wall epithelia, (2) a transition zone where the cells of the epidermis shift towards those of the calicodermis, (3) an undulated base (Fig. 7c,d) and (4) a unique, non-intrusive (< 5 µm), poorly defined calicodermal epithelium which extends from the transition zone and lies under the undulations and initial skeleton that formed on the substrate (Fig. 7e). The lappet-like epidermis contains three cell types; myofibrous epithelial supporting cells, gland cells (some of which are autofluorescent), and nematocysts (Fig. 8). The gastrodermis also contains increased supporting cells but has fewer Symbiodiniaceae than the regular coenosarc. The poorly defined calicodermal epithelium lacked a gastrodermal layer and possessed calicoblasts and inverted anchoring cells, which left outdented nodules on the surface of the incipient skeleton.

**Figure 9:**
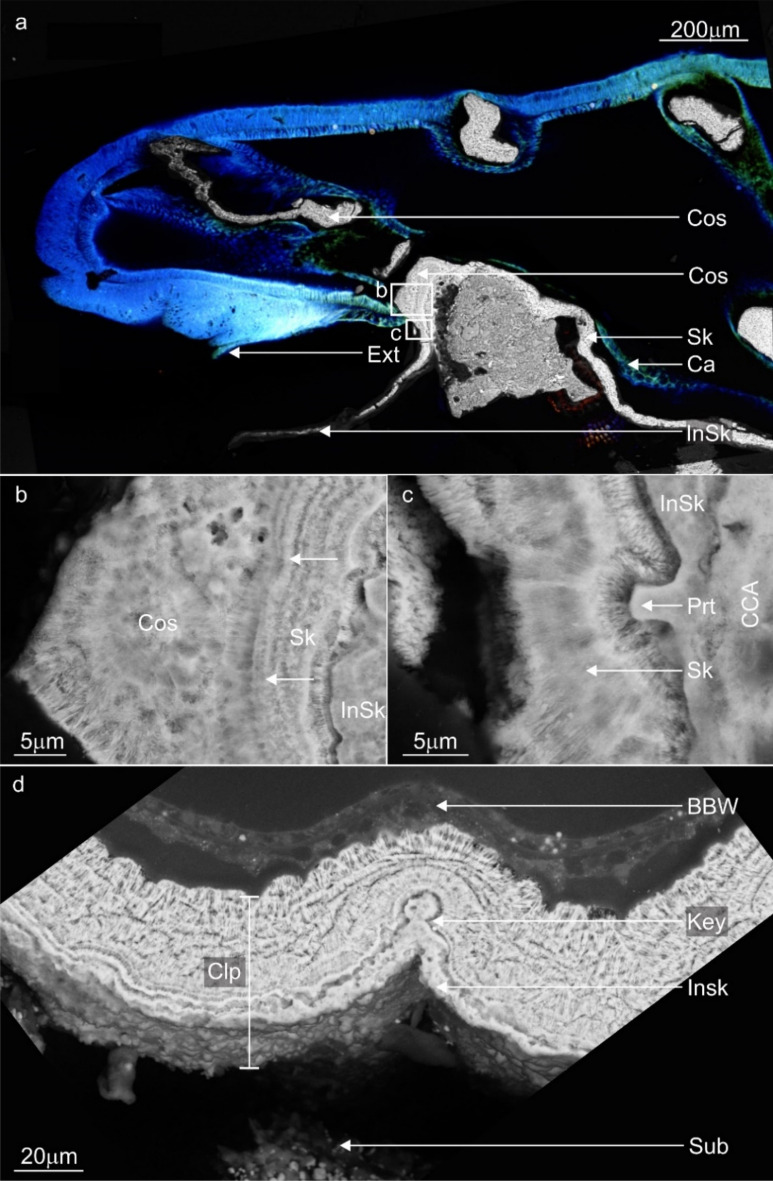
Representative confocal fluorescence microscopy images and backscatter electron microscopy images highlighting the costae wall and initial skeletal layer development in the lappet-like appendage. (**a**) A confocal fluorescence image of the lappet-like appendage overlayed with the corresponding SEM image showing the morphology of the lappet-like appendage and its relationship to the costae (Cos), initial basal deposits (InSk) and the basal skeleton (Sk). (**b**, **c**) First, the lappet-like appendage deposits an initial skeleton (InSk) via the lappet-like appendage poorly defined calicoblastic extension; then, as the lappet-like appendage moves forward, the trailing calicodermis produces a layer of skeleton (Sk) (arrows show the direction of the thickening), building a stronger basal attachment. The lappet-like appendage can generate rapid accretion deposits (RADs) (**b**) that form the costae walls (Cos) and further the robustness of the skeleton/attachment. (**c**) The lappet-like 'appendage's poorly defined calicoblastic extending layer (**a**) (Ext) appears to produce a protuberance (Prt) in the initial skeleton (InSk). (**d**) The smooth initial skeleton layer possesses characteristics similar to those of Clypeotheca (Clp) and can form a keystone structure (Key) in a similar fashion to dissepiments, which can indicate irregular development^8^. CCA; crustose coralline algae, Sub; substrate. calicodermis; Ca, calicodermis extension; Ext, substrate; Sub.

The thin calicoblast layer was closely aligned with the initial skeleton in all samples and was likely responsible for the precipitation of the initial skeleton on the reef substrate (Fig. 7e). The insipient layer of skeleton and subsequent growth layers conform to the substrate's shape (Fig. 9a). The microstructure of the incipient skeleton nearest to the substrate has variable distribution and thickness across the contact interface. It appears to radially extend from numerous single nucleation points most likely points of contact such as calicoblasts (Fig. 9d). The incipient skeletal layer appeared as a low density of composite needle-shaped biocrystals characteristic of secondary skeletal growth and the rapid accretion deposits (RAD), suggesting it to be an organic-rich skeletal layer (Fig. 9b,c). Early costae structures form as a part of secondary growth perpendicular to the substrate (Figs. 7e, 9a). The morphology of the lappet-like appendage may change depending on the presence of costae developing. The emergent costae developed in the lappet-like appendage as rapid accretion deposits (RADs) (Fig. 9a) which also show the direction of the skeletal development. The costae wall nucleated from a 'pocket' of calicoblastic cells (calicoblasts) that developed between the gastrodermis and the epidermis of the lappet-like appendage's SBW (Fig. 7f). This was similar to the proliferation of calicoblastic cells on the gastrodermis after autolysis (phase 2). The 'pocket' calicodermis separates, along with the gastrodermis, from the SBW of the lappet-like appendage as the lappet moves along the substrate (Fig. 7f).

## Discussion

### Phase one: contact response

Initial contact with the substrate is abrasive^1,26^, causing wounds to form in the coral tissues (Fig. 2) as a result of direct tissue contact with foreign materials^1,33,59^, which in turn can cause disease and infection in the fragment^60^. In response to wound formation and foreign material contact, *A. millepora* triggered a localised contact response characterised by mesenterial filament deployment (Fig. 2), cellular proliferation and movement (Fig. 4), as well as mucus development (Figs. 2, 3), which are all phenomena associated with colony protection in reef-building corals, such as *Porites lutea, P. lobata, Acropora aspera, A. pulchra A. polystoma, Montipora patula and Acropora millepora*^28,30,32,57,61,62^. Such a high degree of tissue and cellular plasticity and the speed of mesenterial filament deployment could indicate a highly coordinated signalling response and cellular communication to aid recovery^63–65^. The mesenterial filaments that were deployed possessed several secretory cells capable of sterilisation, digestion and mucus production (Fig. 3c,d,e)^58,66–69^ that, along with surface mucus production via the epidermis, likely helped clean the substrate^70^ such that fragment wounds^65^ experience lowered exposure to disease causing agents and infection that may cause colony mortality.

Histological analysis of the enlarged tissues at the contact interface showed that the bulk of the rapidly proliferating cells were supporting cells during the first two phases. This rapid proliferation and differentiation of cells observed further supports a link between the contact/immune response and locally accelerated growth in reef-building corals^71^. While the supporting cells made up most of the epidermal populations, there was also a suite of secretory gland cells irregularly spread through the tissues (Fig. 4). Previously, only the proliferation of fibroblasts and amoebocytes have been linked to cell proliferation at wound sites in coral^29^. However, our observations suggest that secretory gland cells and supporting cells are also part of this process. Lack of observation for these other cell types^30,57^ as part of the immune response in previous works may partially reflect a lack of histological studies that specifically characterise Scleractinia cells morphology and functional roles.

Gland cells were present at all stages of development in the contact epidermis (Fig. 4)^58,68,72^ and may have aided digestive enzyme development (type 1 gland cells) and mucus development and substrate adhesion (type 2 gland cells)^58,67,68,72^. In particular, the more robust mucus gel/plug that developed at the contact interface can act as a physiochemical barrier to promote beneficial microbes and antimicrobial activity resulting in near-sterile environments^73–75^. This mucus structure is likened to mucus sheets found in *Porites *sp. and plugs that have been observed in anemones^65^. The presence of these gland cells in mesenterial filaments may reflect their digestive capacity and role in substrate sterilisation, mucus production and wound healing^65,70,76^. However, further molecular characterisation of these cells is required to understand the functional diversification more clearly at both cell and tissue levels in the epidermis, lappet-like and mesenterial filaments of *A. millepora*.

### Phase two: soft tissue anchoring and fragment stabilisation

Tissue anchoring was facilitated by the continued proliferation of supporting cells with myofibrils and, subsequently, the cytoskeleton. This process caused the undulations in the tissues^77,78^ and improved the ability for localised inflation and contraction (pulsing). Whilst pulsed inflation is commonplace in most Cnidaria, including Scleractinian corals^46–48,79^ it is rarer or perhaps more discrete in corals due small tissue volumes such as Acropora. Pulsing combined with the undulated tissue morphology of the contact interface SBW ultimately helps anchor the tissue and, subsequently, creates an enclosed space at the interface between the tissue and the substrate that is weakly sealed off from the surrounding seawater (Fig. 5). An enclosed space could serve multiple functional roles, such as allowing contact between the calicoblasts and the precipitation site, producing an enclosed environment required for the extracellular matrix to develop (ECM), developing of adhesive mucus or glycoprotein gels isolated from ambient seawater, and/or supplying an environment for autolysis and the transition from the surface body wall (SBW) into a skeleton producing the basal body wall (BBW). Verifying the enclosed space's roles requires further detailed and targeted investigation.

Deployment of mesenterial filaments to the enclosed tissue interface created by the anchored tissues led to the autolysis of the epidermis (captured by time-lapse microscopy). In most cases, it is likely that the gastrodermis was not removed during this process, as evidenced by patches of the vestigial epidermis. In principle, retaining the gastrodermis would maintain a level of tissue coverage should it become dislodged before the transition is complete, limiting energy expenditure during rapid growth and lessening the time required to establish attachment (phase three).

### Phase three: calcification and the development of a lappet-like appendage leading to substrate encrustation

Phase three of *Am-*CAM begins with the development of an initial skeletal layer and complex lappet-like appendage at the rim of the anchored tissues which, in turn, leads to substrate encrustation by reorganised tissue and eventually secondary skeletal growth. The lappet-like appendage (Fig. 7) is comprised of thicker, highly muscular epithelial layers (Fig. 8) and undulations at its base, which is similar to the undulations that appear in 'lappet's of epithecate coral^5^. These undulations in phase three may also operate similarly to those in phase two^80^ by keeping the surrounding environment out whilst gripping the surface. The semi-permanent attachment would presumably allow for more rapid lappet-like appendage expansion and contraction via the pulsed contractions while moving over the surface without the need to consistently repair or replace attaching cells^52^.

The epithecate lappet is the transition between the SBW and the BBW in the same way as the lappet-like appendage in *A. millepora* is^5,81^. However, in contrast to the epithecate lappet, the lappet-like appendage in *A. millepora* possesses a unique continuation of the calicodermis (located under the undulations at the base of the lappet-like appendage) that is responsible for incipient skeletal production. As the calicodermal extension sat between the lappet undulations and the skeleton or substrate, it's thin nature may have helped it not to interfere with the *A. millepora's* lappet attachment and movement.

Previous iterations of the lappet observed in epithecate corals *Montastrea annularis, Porites astreoides, Gardineria spp.* and *Isophyllia spp.* were incapable of encrusting growth across the reef substrate (horizontal). Instead, it was only capable of *vertical* advance initial basal plate producing an epitheca (outer wall)^5,81^. However, the lappet-like appendage in *A. millepora* is responsible for the horizontal encrustation and basal skeletal advance of the initial and secondary skeleton—expanding the colony size and/or attachment base. This encrusting growth resembles that of clypeotheca development^8^. Clypeotheca is described as epitheca-like skeletal wall that seals over parts of the corallum in areas of stress or damage in modern colonial corals. Although Nothdurft and Webb did not directly observe tissue, they suggest a collaborative process where polyps and adjoining coenosarc seal themselves off from the surrounding environment as they contract and die and the presence of a lappet. The lappet-like appendage observed in *A. millepora* may also perform the hypothesised role in clypeotheca development^8^, providing forward advance of the coral skeleton. The similarities of clypeothecal growth with skeletal attachment in *A. millepora* (Fig. 9) suggest initial skeletal layer helps stave off invasion by parasites and disease while the fragment attaches^8,33^. We have shown for the first time that a lappet-like appendage exists in adult colonial reef-building corals and is responsible for expanding a colony and/or improving stability, allowing for integrated polyp development.

Gland cells formed in the lappet-like appendage that appeared to differentiate from the corals supporting cells. These unique cells developed large gland vesicles at the cell's apical surface which trapped debris similar to zymogen vesicles^67^ or lipopolysaccharide endotoxins that protect the epithelium from bacteria^69,82^. The differentiation of these specialised supporting cells serves two functions; (1) to produce a sterile environment for continued encrustation over a reef surface covered in organisms and potential pathogens and (2) to expand the fragment cytoskeleton via epitheliomuscular development^83–85^ to improve complex movement. In combination, these two factors allow the lappet to produce efficient encrustation of the reef surface without compromising colony health. Such processes highlight the multifunctional capacity of the supporting cells of *A. millepora,* which is similar to the stem cell-like cells found in Hydrazoa^83,86^.

## Conclusions

By observing the attachment process using time-resolved high-resolution imaging across multiple spatial and temporal scales, our study has integrated the behaviour of coral fragments with the ontogeny of their cells and skeleton to provide a novel coral attachment model (*Am*-CAM) for a commonly examined Indo-Pacific coral species *A. millepora*. We have established fundamental biological processes leading to fragment attachment in model species *A. millepora*, elucidating how coral initiate and develop towards successful asexual reproduction and aid reef recovery post-disturbance.

Moreover, unlocking *A. millepora* attachment processes provides a timely means to understand better coral attachment effectiveness, which is a major factor limiting the success of propagation-based restoration^18,21,87^. For example, identifying the key biological factors (across the various stages) that regulate effective attachment and, in turn, the limitations for attachment relative to both biological (taxonomic) and environmental (physical, chemical) features across diverse reef sites. Such knowledge could provide a foundation for developing metrics (e.g. strength of attachment through time) to aid outplanting strategies and optimise return on effort scores for targeted restoration sites^55,56^. However, for this knowledge to help, this model currently derived from a single species will need to be validated across a broader range of coral species.

## Methodology

### Coral collection and aquaria experiments

Five independent colonies of the reef-building coral *A. millepora* (Ehrenberg, 1834) sourced from coastal waters (16.9186° S, 145.7781° E) off the northern Great Barrier Reef, Australia (purchased from Cairns Marine*,* Cairns, Australia) were acclimated together for one month in a 500-L closed system aquaria at the Queensland University of Technology (QUT). Although the five colonies were not genotyped and so could reflect members of a single clone, we do not expect marked deviation from the attachment behaviour. Tropic Marin Pro^©^ salt-based seawater was maintained at 25 (SD =  ± 1 °C) and 1.023–1.025 sg (specific gravity). Water flow was maintained at 200 L h^−1^ using a wave-maker (Tunze, Germany). Light was delivered via Radion LED units (Ecotech, Pennsylvania, USA) on a 12 h:12 h light: dark cycle peaking in intensity around midday (4 h) at 200 µmol m^−2^ s^−1^, measured at the coral surface using an underwater quantum flux reader (Apogee, Utah, USA). A biological sump of Marine Pure™ (CerMedia, New York, USA), *Caulerpa* sp*.* and coral rubble together maintained inorganic nutrient concentrations (NO_3_^+^, PO_4_^3−^, NH_4_^+^) at very low to undetectable levels (< 0.1 ppm), as monitored by Red Sea^©^ multitest titrations every 3 days. CaCO_3_ (Ca) concentration was maintained between ca. 400–420 ppm through the addition of calcium chloride (CaCl_2_; Seachem Laboratories, Georgia, USA) via micro-dosing (< 5 ml) using an automated system. This was to avoid over/under saturation of calcium (Ca), magnesium (Mg) or alkalinity (KH), keeping the aquaria system chemistry in line with previous studies^88–90^ and calcification rates steady. Salinity was maintained at 34 PSU and monitored daily with a digital refractometer (Hanna Instruments, Woonsocket, USA). The carbonate alkalinity and magnesium concentrations were determined by Red Sea (Red Sea, Herzliya, Israel) multitest titration and maintained between 125 and 150 ppm and 1280 (SD =  ± 50 ppm), respectively.

**Figure 10:**
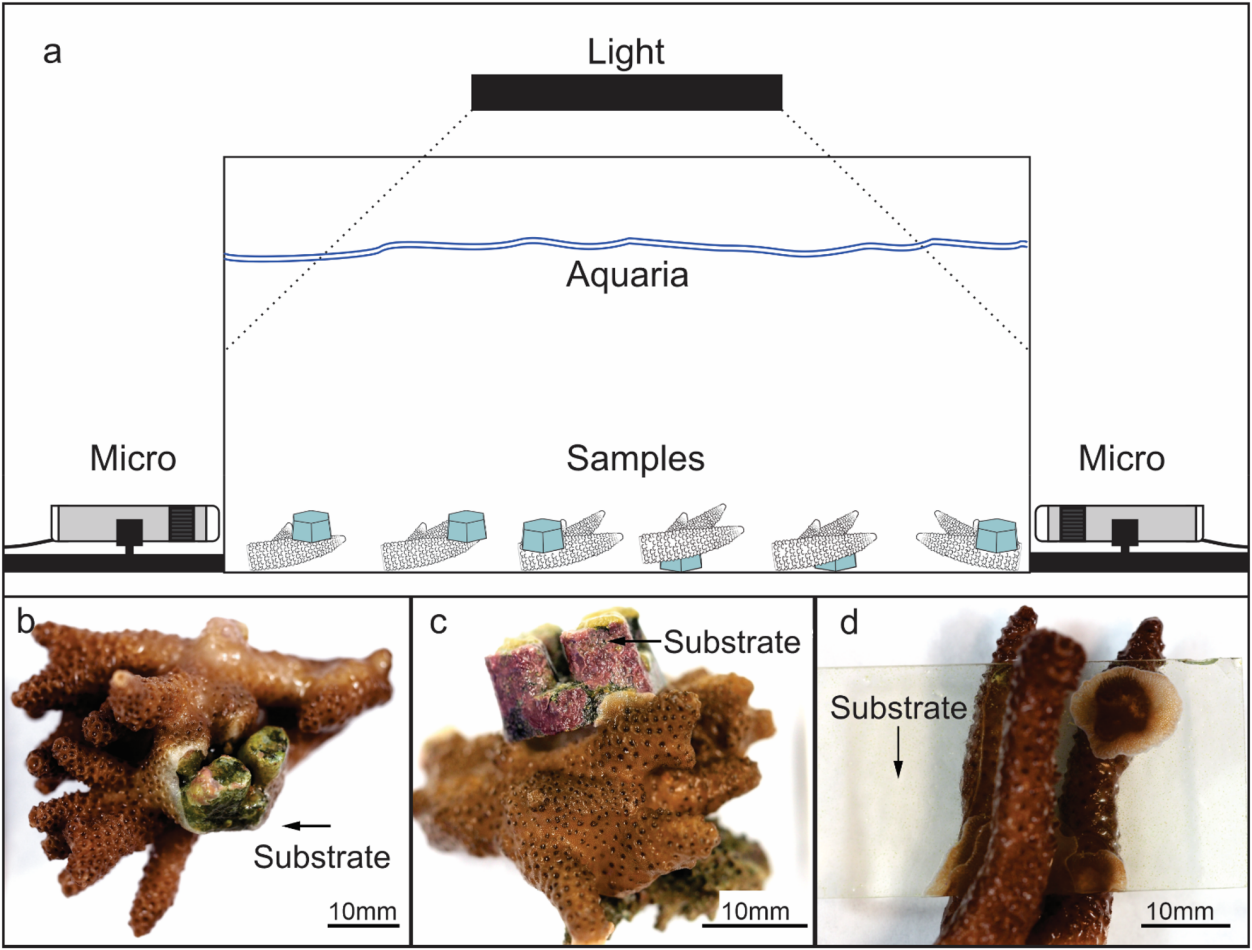
Digital graphic of the aquaria setup for the time-lapse and macro images of the ceramic clast and the glass substrate in the fragments. (**a**) The interface between the coral fragment tissue and the substrate was recorded (time-lapse) in a research aquaria setup with LED light for 30 days using light microscopes. (**b**, **c**) The ceramic substrate was placed either between the branches or at the surface/base of the fragment to maximise contact with both the higher growth areas and slower growth base tissues. (**d**) The glass substrate was lighter and less hydrodynamic than the ceramic, which made it easier to dislodge via water flow and therefore had to be placed between the branches for added stability.

Following the acclimation period, each colony was divided into fragments (*n* = 30), each retaining 3 to 10 branches with a maximum fragment length and width of 7 cm, using a Dremel Cordless Rotary Tool (Bosch, USA). All fragments were acclimated for an additional two weeks in a separate, closed, 50-L experimental aquaria (Fig. 10a) maintained in an identical manner as for the original 500-L holding tank (above) to allow recovery from fragmentation. All fragments were maintained on the glass bottom of the tank and gently repositioned every 2 days to ensure that no fragments began attachment prior to the start of experiments. Chemically inert and pH neutral kiln-fired (2000 °C) ceramic plugs (Ecotech) of consistent size (12 mm × 12 mm × 12 mm), shape (hexagonal) and surface texture (i.e., surface rugosity and shape) were used as substrates to avoid the physical (i.e., irregular surfaces and surface angles) and environmental variables (e.g., biofouling, physical barriers) that may affect the initial attachment processes when using rubble or live rock.

Once the coral fragments were placed in contact with the ceramic substrate, all unnecessary movement or interactions were avoided—including attempts to maintain the sterile surface. As a result, biofouling (e.g. coralline algae, cyanobacteria etc.) on the ceramic surface exposed to light outside of the initial contact area was common and unavoidable. Opaque black ceramic was used to improve time-lapse imaging by limiting areas of high exposure caused by the standard white surface.

An additional five trials were conducted using clear glass slides as a substrate to view the internal physiology of the fragments that were otherwise obstructed by the opaque ceramic plug. Time zero was considered the first point of contact of coral with the ceramic substrate. In nature, the orientation of fragments that fall from parent colonies onto the surrounding substrate is random. Therefore, the substrate was positioned either between the branches (Fig. 10b,d) or at the surface/base of the fragment (Fig. 10c), ensuring contact with both the higher growth apical tips and slower growth base tissues and a fixed upward orientation for axial polyps. The attachment timing between the ceramic and glass substrate did not appear to differ significantly.

### Time-lapse microscopy

Time-lapse microscopy was used to observe processes on the outside of the coral, including soft tissue behaviour and gross anatomy, during the experiment using a AM7915 portable light microscope (Dino-Lite, New Taipei City, Taiwan). Specimens observed via time-lapse were positioned close to the glass walls of the aquaria and within the focal distance of the microscopes situated on the outside of the aquaria (30 mm to 50 mm). The microscopes examined the ceramic plug, glass, and coral surface interface. Images were captured every 2 min for 28 days, with Playback set at 15 frames per second. To determine mesenterial filament density changes over 7 days, an image (i.e., a screenshot) was collected from glass slide time-lapses at an approx. sunrise, midday and sunset. The mesenterial filaments present at the contact interface and external of the body cavity were traced and analysed using ImageJ (NIH, USA) to determine the mean raw surface area of the mesenterial filaments over the total image surface at each time point. Not all videos were able to be analysed due to movement in the camera, sample movement and minor but inopportune data loss due to microscope software failure. Linear and equal adjustments in contrast and brightness were applied to all images described in this manuscript.

### Sample preparation for autofluorescence and electron microscopy

Scanning electron microscopy (SEM) and confocal autofluorescence methods were used for histology and skeletal imaging. Two specimens from the pool of fragments were removed from the aquaria every 3 days over the 28-day experimental period to be dehydrated and embedded in resin. Sample preparation was adapted from previous work^53,91,92^ to observe coral tissue and skeleton as well as the ceramic plug in one section. Samples were fixed in a solution comprising 3% paraformaldehyde, 3% glutaraldehyde and 94% saltwater. Each fixed sample was then placed into 0.1 M sodium cacodylate (C_2_H_7_AsO_2_) solution before being postfixed with both 1% osmium tetroxide (OsO_4_) to as a stain contrast for imaging and ruthenium red (Cl_6_H_42_N_14_O_2_Ru_3_) to fix mucopolysaccharides. Samples were then dehydrated and embedded in Spurrs resin according to the Pelco Biowave osmium staining and embedding protocol and protocols modified from McCutcheon et al. (2018) specific to this study (Supplementary Table S1). Samples were then placed in an oven and left at 60 °C to polymerise for 3 to 7 days (where the rate of polymerisation for any given sample was dependent on the volume of resin). Initial attempts to dehydrate the samples using standard biowave practices did not remove all moisture from the samples, which in turn caused moisture pockets to form in the resin during polymerisation. We tripled the dehydration steps in response and added an additional acetone dehydration phase (Table S1).

Once set, the resin blocks were cut to expose a cross-section of the intrusion/coral interface using a diamond saw. The resin blocks were 10 mm to 20 mm in thickness, allowing additional polishing to produce more data if surfaces were damaged. Cross-sectioned surfaces of fixed and embedded tissue, coral skeleton and ceramic plugs were polished using 3 M wet/dry sandpaper (CAMI, from 400 to 2000 grit), followed by a lapidary polishing wheel and 1 µm aluminium oxide compound on a cloth pad. Samples were then rinsed using deionised water (DI) and placed into an ultrasonic cleaner to remove fine particles from the surface. In the case of SEM, samples were etched in 1% formic acid solution for ~ 10 s before rinsing in DI to expose the skeletal microstructure^93^.

### Scanning electron microscopy

Tissue morphology, cells and skeletal microstructure were imaged using a Zeiss Sigma Field Emission SEM (Zeiss, Oberkochen, Germany). Samples were imaged using 'Zeiss's backscatter detector mode (BSE) under a variable pressure vacuum at 20 kV. The SEM facilitated microanatomical analysis identified the fragments, typical and atypical cell populations, soft tissue morphology, the presence and absence of tissues and skeletal microstructure and development. Using our sampling methods for electron microscopy, individual sites on a single sample (*n* = 25) could be imaged dozens of times but this was highly dependent on the intensity of the beam. The higher intensity beams lead to a degradation of the tissue and cells. If damage occurred, the sample sites could be re-polished to expose new tissue for imaging. Overall, 724 images were produced over 24 samples.

### Autofluorescence microscopy

The micro-autofluorescence of the coral fragments embedded in resin were observed using an A1R HD25 confocal autofluorescence microscope (Nikon, Tokyo, Japan). Excitation wavelengths of 405 nm (DAPI), 488 nm (FITC), 561 nm (TRITC) and 640 nm (Cy5) were delivered using a N4S 4-laser unit. Emission bandwidths were 425–475 nm (cyan autofluorescence protein), 500–550 nm (green autofluorescence protein), 500–620 nm (red autofluorescence protein) and 663—738 nm (near infrared). Laser power was set at 1 to 1.5 for 10 × to 20 × magnification and 2–4 for 40 × to 60 × magnification. The gain was set between 90 and 115 depending on the magnification for cyan, green and red emissions and between 115 and 140 to image the weaker near-infrared emission. Samples that were only stained with Osmium and ruthenium red were unable to be imaged by fluorescence microscopy. In some cases, embedded samples without staining and long laser dwell times damaged the image surface where the resin was poorly cured or soft. However, this can was resolved with an additional round of polishing. Overall, 127 images were produced over 17 samples.

## Supplementary Information

Supplementary Information 1.

Supplementary Video 1.

Supplementary Video 2.

Supplementary Video 3.

Supplementary Video 4.

Supplementary Video 5.

## Data Availability

All data needed to evaluate the conclusions in the paper are present in the paper and/or the Supplementary Materials. The datasets used and/or analysed during the current study are available from the corresponding author on reasonable request.
