# Supplementary material for: Cellular adaptations leading to coral fragment attachment on artificial substrates in Acropora millepora (Am-CAM)
Source: Sci Rep. 2022 Nov 1;12:18431. doi: 10.1038/s41598-022-23134-8 (PMC9626494; doi:10.1038/s41598-022-23134-8)
Supplement: Supplementary file 1 — Supplementary Information 1. [file 41598_2022_23134_MOESM1_ESM.docx]

SUPPLIMENTARY MATERIAL

Lewis, B.M., Suggett, D.S, Prentis, P.J., & Nothdurft, L.D. (2022). Attachment of Acropora millipora fragments to a substrate: a model for mature coral attachment (Am-CAM). Scientific Reports.

Supplementary Table S1: The protocol post-fix, staining, dehydration and embedding (EPON) for sample preparation of the coral fragment fixed in a 3% solution of glutaraldehyde and 3% paraformaldehyde using the *Pelco Biowave* to view with SEM and confocal autofluorescence microscopy. Dehydration and infiltration steps can be added/removed depending on the sample's size and permeability. The osmium step was removed for the confocal autofluorescence microscopy.

| **Processing Phase** | **Microwave Setting (Watts)** | **Time of Procedure (seconds)** | **Under vacuum in Microwave (x psi)** |
| --- | --- | --- | --- |
| **Buffer Rinse** |  |  |  |
| 0.1 M Sodium Cacodylate | - | 10 minutes | No |
| 0.1 M Sodium Cacodylate | 80 | 40 s | No |
| **Post Fixative and Staining** |  |  |  |
| 2 x Osmium Tetroxide (x%) + Ruthenium Red (x%) solution | 80 | 120 s interval | Yes |
| **Rinse** |  |  |  |
| 2 x DI water | 80 | 40 s | No |
| **Dehydration (% Ethanol Solution)** |  |  |  |
| 3 x 20% repeated with 10% increments to %100 (70% and 100% left overnight) | 250 | 40 s | No |
| **Dehydration (% Acetone)** |  |  |  |
| 3 x 100% (left overnight) | 250 | 40 s | No |
| **Resin infiltration (% Spurrs Resin in Acetone)** |  |  |  |
| 15% | 250 | 180 s | Yes |
| 30% | 250 | 180 s | Yes |
| 50% | 250 | 180 s | Yes |
| 70% (left overnight then microwaved) | 250 | 180 s | Yes |
| 90% | 250 | 180 s | Yes |
| 3 x 100% (left overnight) | 250 | 180 s | Yes |

Supplementary Table S2: shows the initiation timing and duration of the behaviours in *A. millipora* samples (n=5) (Fig. 1).

|  | Initiation average (Days) | STD | Duration average (Days) | STD |
| --- | --- | --- | --- | --- |
| **Wounds present** | 1 | 0 | 2 | 0.71 |
| **Soft tissue enlargement** | 1.8 | 0.4 | 6 | 3.54 |
| **Mesenterial filament activity** | 1.25 | 0.43 | N/A | N/A |
| **Mucus secretion/present** | 1.25 | 0.43 | N/A | N/A |
| **Soft tissue anchoring** | 5 | 2.28 | 9 | 1.9 |
| **Autolysis** | 4 | 0.71 | 4 | 4.98 |
| **Calcification** | 11 | 1.79 | N/A | N/A |
| **Lappet develops** | 8.2 | 2.23 | N/A | N/A |
| **Encrustation begins** | 11.2 | 2.14 | N/A | N/A |

Supplementary Figure F1: Table highlighting the mean mesenterial filament density at the contact interface and external to the body cavity compared to the initial contact surface area over the first seven days of time-lapse microscopy on specimens in contact glass slide viewed from the underside. The Y axis represents a ratio percentage area of mesenterial filaments observed normalised to the area of the initial contact lesion at the point of contact between the specimen and glass slide. 1-4 represents the number of replicates.

**[Supplementary Movie S1 (.Mp4 format).](https://cloudstor.aarnet.edu.au/plus/s/FrKSIMLEjBMjQp1)**[This video shows the peak mesenterial filament behaviour over four days of the coral’s contact response as seen through a glass substrate (Fig. S2). During this time, the tissue wounds heal, the soft tissues become enlarged, mucus is expelled, creating a biofilm, and ciliates and other colonisers populate the mucus biofilm.](https://cloudstor.aarnet.edu.au/plus/s/FrKSIMLEjBMjQp1)

**[Supplementary Movie S2 (.Mp4 format).](https://cloudstor.aarnet.edu.au/plus/s/BfNqfQQRuG2oE6h)**[The mesenterial filaments twist in a corkscrew motion to move through cinclide-like temporary holes in the coral fragment’s soft tissues, gaining access to the substrate outside of the coral fragment’s body (Fig. S3).](https://cloudstor.aarnet.edu.au/plus/s/BfNqfQQRuG2oE6h)

**[Supplementary Movie S3 (.Mp4 format).](https://cloudstor.aarnet.edu.au/plus/s/26fqXsXpfvNHKyK)**[Enlarged tissues at the contact points between the coral fragment and a ceramic substrate developing and anchoring onto the substrate (Fig. S2).](https://cloudstor.aarnet.edu.au/plus/s/26fqXsXpfvNHKyK)

**[Supplementary Movie S4 (.Mp4 format).](https://cloudstor.aarnet.edu.au/plus/s/GzjFJRNwvOEXRLX)**[The mesenterial filaments remove the coral's epidermal tissues or SBW via autolysis (Fig. S6) and in some cases, the gastrodermis can still be seen intact.](https://cloudstor.aarnet.edu.au/plus/s/GzjFJRNwvOEXRLX)

**[Supplementary Movie S5 (.Mp4 format).](https://cloudstor.aarnet.edu.au/plus/s/9lyORcSuAcGRqTw)**[This video time-lapse highlights the localised pulsing of the lappet-like appendage as it slowly moves across the surface, depositing the coral’s initial skeleton (Fig. S7).](https://cloudstor.aarnet.edu.au/plus/s/9lyORcSuAcGRqTw)
